# A process mineralogy approach to study the efficiency of milling of molybdenite circuit processing

**DOI:** 10.1038/s41598-020-78337-8

**Published:** 2020-12-03

**Authors:** Ataallah Bahrami, Morteza Abdollahi, Mirsaleh Mirmohammadi, Fatemeh Kazemi, Abolfazl Danesh, Maryam Shokrzadeh

**Affiliations:** 1 Department of Mining Engineering, Faculty of Engineering, Urmia University, P.O. Box 57561/51818, Urmia, Iran; 2 School of Mining Engineering, College of Engineering, University of Tehran, Tehran, Iran; 3 Faculty of Engineering, University of Kashan, Kashan, Iran; 4 Complex of Copper Processing-Sungun, East Azerbaijan Province, Headquarters Rd, Tabriz, Iran

**Keywords:** Energy science and technology, Engineering, Materials science

## Abstract

This study is conducted with the aim of investigating the efficiency of open and closed-circuit molybdenite ore comminution processes (primary and secondary mill, respectively), through mineralogical study of mills feed and product. For this purpose, particle size distribution, minerals distribution, degree of liberation and interlocking of minerals in mills feed and product were studied. According to the results, chalcopyrite, molybdenite, pyrite and covellite constitute the major part of the mineral composition of open-circuit mill feed. Minerals at the mill product, in the order of abundance include liberated molybdenite particles, liberated chalcopyrite and interlocked chalcopyrite with pyrite, liberated and interlocked pyrite particles, and associated silicate gangues. The d_50_ values of the feed and product particles of the open-circuit mill are equal to 13.80 and 13.40 microns, respectively. Degree of liberation of molybdenite for the feed and product of this mill is almost the same and is equal to 98.0%. Closed-circuit mill feed includes, in order of is abundance, liberated molybdenite particles in the form of blades and irregular polygonal shapes, liberated and interlocked chalcopyrite, and liberated and interlocked pyrite particles with gangue minerals. Molybdenite particles in the mill product are almost completely liberated, and the degree of liberation values of chalcopyrite and pyrite are 84.40% and 91.40%, respectively. According to particles size distribution of the feed (d_50_ equal to 25.03 microns) and the product (d_50_ equal to 24.24 microns) of closed-circuit mill, it can be stated that comminution is not well-operated in closed-circuit mill due to the low solid percentage of closed-circuit mill feed and the inefficiency of hydrocyclone. Examination of Mo, Cu, and Fe grade variations for 10 days in both off and on modes of mill shows that closed-circuit mill does not have an impact on comminution process. It can even be concluded that the mill has a destructive effect the flotation process by producing slimes.

## Introduction

Liberation of valuable minerals from associated gangue minerals is an important and fundamental step in separating an ore mineral from gangue during physical or physicochemical separation processes. Crushing and grinding processes are typically used by crushers and mills to liberate minerals, which are energy-intensive processes (especially fine grinding by mill). Meanwhile, ball mills are known for their lowest energy efficiency. The efficiency of ball mills is about 1.0% and, in some cases, less than 1.0% based on energy consumption.

Mineral comminution theories are often based on the relationship between the size of the primary feed particles entering the mill and the energy consumed (Eq. 1); in most of these relationships, it has been assumed that the ground material is brittle^1^. In other words, the grindability indices of the minerals in a deposit are typically used for designing a comminution circuit for an ore. These indices are proposed with the assumption of homogenic breakage and continuity of the ore, regardless of the textural properties of the minerals on a micro scale^2^.

1 $$dE=-k\frac{dx}{{x}^{n}}$$

In Eq. (1), dE is the specific energy for grinding (kwh/t), k is the grinding constant, and dx/x^n^ is the variations of particle size during grinding.

The main missing factor in most comminution theories is the relationship between ore grinding and its textural and mineralogical characteristics. Each ore in the mine has different geomechanical characteristics for various reasons, such as the effect of faults, the presence of dikes, as well as the type of the deposit in different zones of the mine (such as oxidized, supergene and hypogene zones). Variations in geomechanical characteristics will cause different comminution behaviors for ore during blasting and comminution operations by crushers and mills. In other words, due to the diversity of minerals and the difference in their grindability indices, the feed of processing plants includes a distribution of mineral types and particle sizes. Therefore, the study of mineralogy and the textural characteristics of an ore and its host rock will provide valuable information from the perspective of ore comminution behavior and minerals content and thus the design or optimization of their comminution processes.

Ore textural parameters including hardness, minerals liberation degree, particle size, particle size distribution, minerals abundance, type of minerals, interlocking between minerals and mineralogical structures are important in the issue of ore grindability^1,3,4^, and can be considered as optimizing parameters for comminution processes. Many researchers^1,5–8^ have extensively investigated the impact of textural parameters of ores in mineral processing. It should be noted that the combination of these parameters with the operating conditions of concentration processes, especially comminution, is very complex due to the randomized mechanism of breakage in mills. In general, the process mineralogy of the feed and product of comminution process (mill) will lead to presenting solutions to optimize the operation of the existing circuit and corrective suggestions in the flowsheet. In other words, mineralogy of the comminution process leads to the determination of the optimal conditions of the process by providing practical information on the liberation degree of valuable minerals in each size fraction, particle size distribution and how the minerals are interlocked^9^.

**Figure 1:**
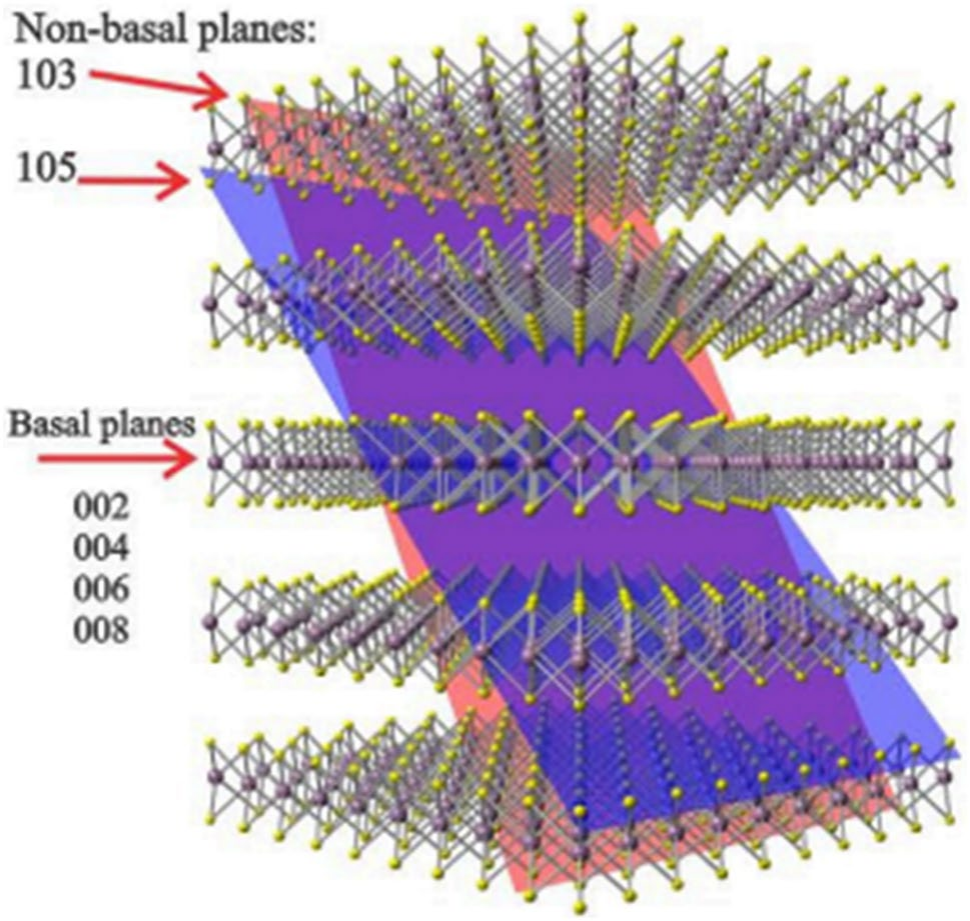
Image of the crystalline structure of molybdenite mineral^13^.

Molybdenite is a sulfide mineral and is often found in copper-molybdenum porphyry deposits, which is processed as a by-product of copper during the flotation process^10^. Molybdenite mineral is one of the most stable members of Transition Metal Dichalcogenides (TDMs), which are present in the hexagonal system as a 2H poly as well as a 3R. Figure 1 shows a schematic image of the MoS_2_ layered structure along with the XRD pattern crystallographic plates. Sulfur atoms at higher and lower surfaces surround smaller molybdenum atoms in the form of sandwiches^11^. Molybdenum and sulfur atoms inside the layers are bonded together by strong covalent bonds, but the successive layers of sulfur atoms are joined bonded by weak Van der Waals bonds^12^.

Due to the crystalline structure mentioned, molybdenite has anisotropic properties, which has led to its different behavior in different faces^14^, including the fact that the anisotropic property leads to the preferred orientation of molybdenite mineral during grinding; and as the particle size decreases, this orientation increases. In this way, molybdenite is broken under grinding at two different surfaces. Surfaces created by breaking S–S bonds (non-polar surfaces) and surfaces resulting from the breakage of strong Mo-S bonds (polar edges)^15^. It is worth mentioning that in the structure of molybdenite, the bond between S–S and Mo-Mo is of Van der Waals type and S–Mo–S is of covalent type. Due to the fact that Van der Waals forces/bounds are relatively weak compared to covalent bonds, it is more likely to break at the edges. On the other hand, the behavior of molybdenite in other concentration processes, such as flotation, is greatly influenced by how it breaks and its liberation degree during milling. The natural floatability of molybdenite is related to its textural characteristics such as flatness, roundness of particles, longitudinal elongation ratio and smooth surfaces. Due to the preferred cleavages along weak S–S and Mo–Mo bonds during the grinding process, plate-like fragments are produced from larger particles. Flat and long particles cause poor performance of particle-bubble attachment and thus reduce recovery. Therefore, molybdenite flotation behavior is the result of a combination of the property of natural floatability and particle morphology.

The study of the flotation of copper and molybdenite ores indicates that the recovery of molybdenite and copper flotation is reduced in the coarse, fine and very fine size ranges of these minerals. The highest recovery of molybdenite and copper occurs at sizes 27–55 microns^16^, therefore the optimal grinding of these minerals is of great importance. Due to the anisotropic behavior of molybdenite and its association with other sulfide minerals (and other associated gangue minerals), performing process mineralogy studies can lead to results for proper design or optimization of its comminution circuit. In the present study, the type and behavior of copper sulfide, molybdenite and associated gangue minerals, especially pyrite, have been identified through a process mineralogy approach toward molybdenite comminution circuit (Sungun copper-molybdenum processing complex located in northwestern of Iran). For this purpose, mineralogical studies have been performed on mill feed and product. As a result of these studies, the liberation degree and the particle size, distribution and the interlocking mode of the minerals have been determined. Analysing and combining this information with the operating conditions of the plant led to solutions for optimizing the current comminution circuit. In other words, according to the mineralogy of feed and product of mills, the most optimal operating conditions are determined and implemented in order to improve the efficiency of the circuit.

## Materials and methods

### Studied concentration and comminution circuit

**Figure 2:**
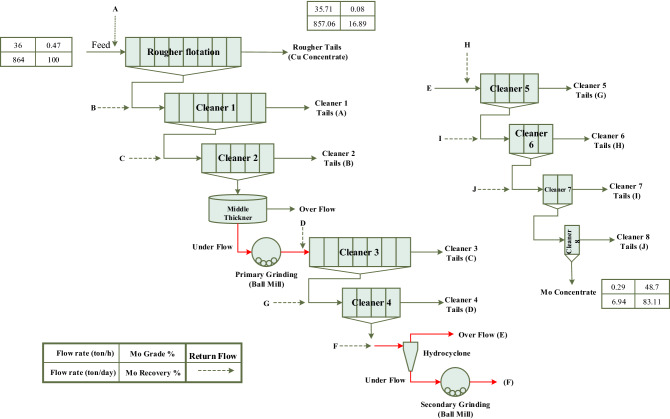
A schematic of the molybdenite processing circuit of the Sungun copper–molybdenum processing plant.

The present study was performed on the grinding efficiency of molybdenum flotation circuit mills of Sungun copper—molybdenum processing complex. Sungun copper mine and complex with geographical coordinates of 46˚ 43ˈ east and 38˚ 42 ˈ north is located in northwest of Iran. In the molybdenum processing plant of Sungun complex (flowsheet is shown in Fig. 2) uses two ball mills to perform grinding operations. The primary ball mill operates in an open circuit (after the middle thickener) and the secondary ball mill operates in a closed circuit with a hydrocyclone. The underflow of the middle thickener with a solid percentage of about 55.0% enters the primary ball mill, and the mill product enters the cleaner 3 flotation cells after dilution. The length and diameter of the ball mill used in this department are 2.44 and 1.52 m, respectively, which has a slurry mass capacity of 4.13 t/h. Cleaner 4 concentrate is introduced into hydrocyclone clusters (two hydrocyclone clusters, each of which consists of 3, 6-in. cyclone devices) and is separated into a fine overflow fraction with particles size smaller than 38.0 microns and an overflow fraction with particles size larger than 38.0 microns. The overflow of each cluster is transferred to 5–8 cleaner cells and its underflow enters a regrinding ball mill, which is in a closed circuit with these hydrocyclones. The goal of the regrinding stage is to achieve the maximum liberation degree of molybdenite and copper minerals and their liberation from each other. The length and diameter of the closed-circuit ball mill are 1.83 and 1.22 m, respectively, which has a slurry mass capacity of 3.6 t/h and a solid percentage of 50–60% (designed for the plant).

In order to study the mineralogy of milling process in the molybdenum processing circuit, samples of feed (middle thickener underflow) and product of open-circuit ball mill, as well as overflow and underflow of hydrocyclone (feed) and product of closed-circuit ball mill were prepared. Sampling points are marked in red in Fig. 2. It is worth mentioning that 3 samples were collected using a sampling spoon from each location and at 30-min intervals, (to investigate the effect of plant feed variations). After filtering and drying, 30 g of the sample was prepared using a riffle sample splitter to check size distribution, preparation of polished sections and performing microscopic studies.

### Particle size distribution (PSD) of feed and product of open and closed-circuit ball mills

**Figure 3:**
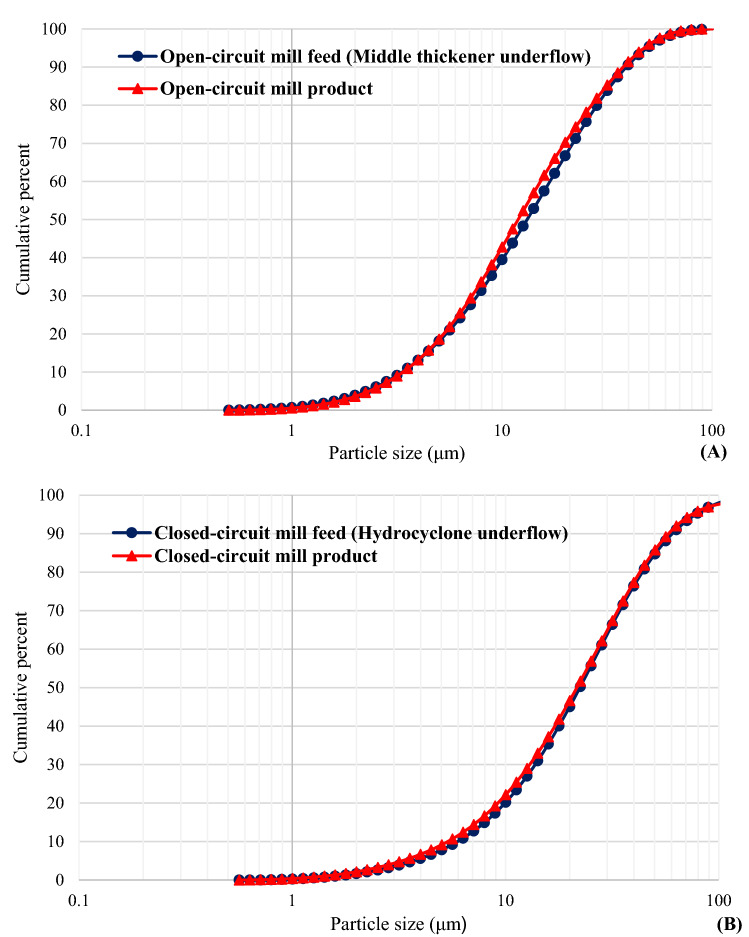
Particle size distribution (laser particle size analyzer) of feed and product of **(A)** open-circuit ball mill and **(B)** closed circuit ball mill (Sungun copper–molybdenum complex).

In order to perform the process mineralogy studies on the samples, optical microscopic studies were performed on polished sections after ehaviour the size of the feed and product particles using Laser Particle Size Analyzer (SLS: mastersizer 2000/Malvern Panalytical technology). The results of particle size analysis of feed and product samples from grinding circuits are shown in Fig. 3, and comparison of their d_10_, d_50_ and d_90_ values are performed in Table 1. According to the results, grinding by open-circuit ball mill has caused the particles size to decrease from 90.0% smaller than 45.0 microns to about 99.0% smaller the 45.0 microns. Grinding by closed-circuit ball mill has also reduced d_90_ value of particles from 67.96 (mill feed) to 65.13 microns (mill product).

**Table 1:** Comparison of d_10_, d_50_ and d_90_ values of feed and product of open and closed-circuit ball mills (Sungun copper–molybdenum complex).

Sample		d_10_ (μm)	d_50_ (μm)	d_90_ (μm)
Open-circuit ball mill	Feed	3.76	14.79	43.83
	Product	3.80	13.40	42.33
Closed-circuit ball mill	Feed	6.68	25.02	67.95
	Product	6.02	24.23	65.13

### Mineralogical studies

Microscopic study of polished sections is the most common method of studying the mineralogical properties and textural association between minerals in mineral samples. In order to investigate the grinding ehaviour of the minerals in the molybdenum processing circuit, microscopic studies were conducted on polished sections prepared from the collected samples. Microscopic studies were performed using the Leitz polarizing microscope of model SM-LUX-POL equipped with a digital imaging camera at the college of Mining Engineering, University of Tehran. Based on the results of mineralogical studies, open-circuit mill feed contains chalcopyrite, molybdenite, pyrite and covellite. Besides, molybdenite, chalcopyrite, and pyrite are the major minerals that make up the feed of closed-circuit mill (hydrocyclone underflow). Table 2 shows the important physical/chemical properties of various minerals in the feed of open and closed-circuit mill of the molybdenite processing circuit.

**Table 2:** Selective physical/chemical properties of sulfide minerals in open and closed-circuit mills feed—molybdenum processing plant (Sungun copper—molybdenum Complex).

Mineral	Cleavage	Hardness (Mohs scale)	Tenacity	Chemical bonding	Breakage mode
Molybdenite	Perfect on {0001}	1–1.5	Flexible	Van der Waals/ Covalent	Plate-like
Chalcopyrite	Poor/indistinct	3.5–4	Brittle	Ionic to Covalent	Irregular shape
Pyrite	Poor/indistinct	6–6.5	Brittle	Ionic to Covalent	Irregular shape
Covellite	Perfect on {0001}	1.5—2	Flexible	Van der Waals/ Covalent	Semi plate-like

## Results and discussion

### Investigating the efficiency of open-circuit ball mill from process mineralogy perspective

#### (A) Particle size distribution study

**Figure 4:**
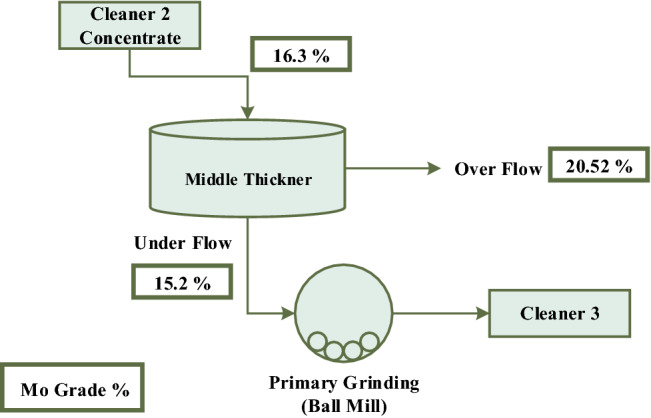
Molybdenite grinding circuit—open-circuit ball mill.

The feed of the primary ball mill or the open-circuit mill is the underflow pulp from the middle thickener (Fig. 4). According to Fig. 2 circuit, the middle thickener with a diameter of 12 m and free settling mechanism (in the molybdenum plant of the Sungun copper–molybdenum Complex) is located after cleaner 2 and before the open-circuit mill. The feed pulp to this thickener has a solid percentage of 13.87, which after settling, the underflow is discharged with a solid percentage of about 60.0% and the overflow weight percent is 0.04%. Based on the grade analysis performed on the underflow of the thickener or open-circuit mill feed, the grade values of Mo, Cu and Fe elements are 23.71, 17.57 and 14.84%, respectively. Due to the 23.71% value of molybdenum grade, this product cannot be supplied as a final concentrate and it is necessary to perform more processing stages (cleaner flotation stages). Therefore, the purpose of grinding at this stage is to achieve more liberation of copper minerals from molybdenite.

**Figure 5:**
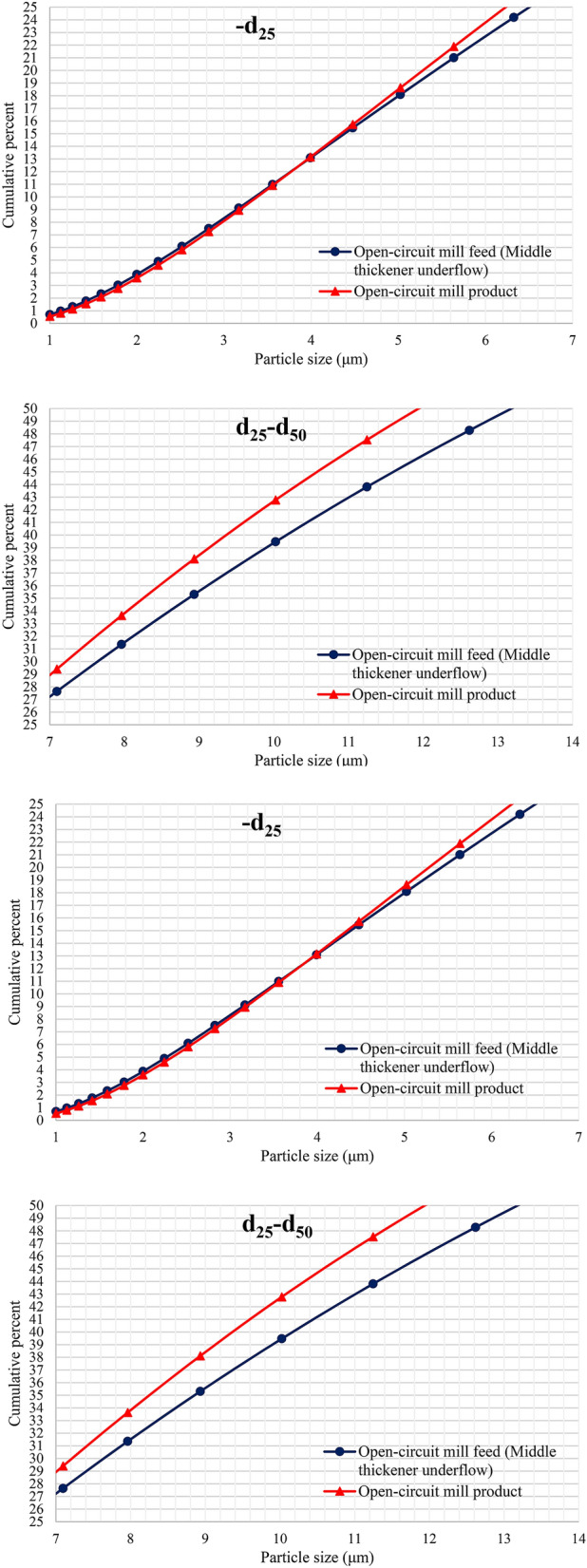
PSD diagrams of feed and product of open-circuit mill.

According to the PSD diagram shown in Fig. 5 as well as Table 1, the grinding process in open-circuit mill produces about 70.0% of the fine product with particle size smaller than 20.0 microns; of this amount, 40.0% is smaller than 10.0 microns in size. Particles with a size smaller than 7.0 microns also have a significant volume and account for approximately 25.0% of the mill product particles. Based on the results, it can be concluded that the highest amount of grinding occurred for particles in the size ranges of d_75_-d_25_ of feed. Grinding also occurred for feed particles smaller than d_25_ (7.0 microns), but grinding did not have a desirable result for particles larger than 23.0 microns. Given that the product of open-circuit mill is the feed to cleaner 3 flotation cells, the size distribution of the mill product particles (in other words, the amount of grinding in the mill) is of great importance. Since with increasing the amount of particles smaller than 10.0 microns, flotation recovery of molybdenite gradually decreases due to reduced probability of collision and attachment to air bubbles ^17^.

#### (B) Mineralogical study of the open-circuit ball mill feed and product

**Figure 6:**
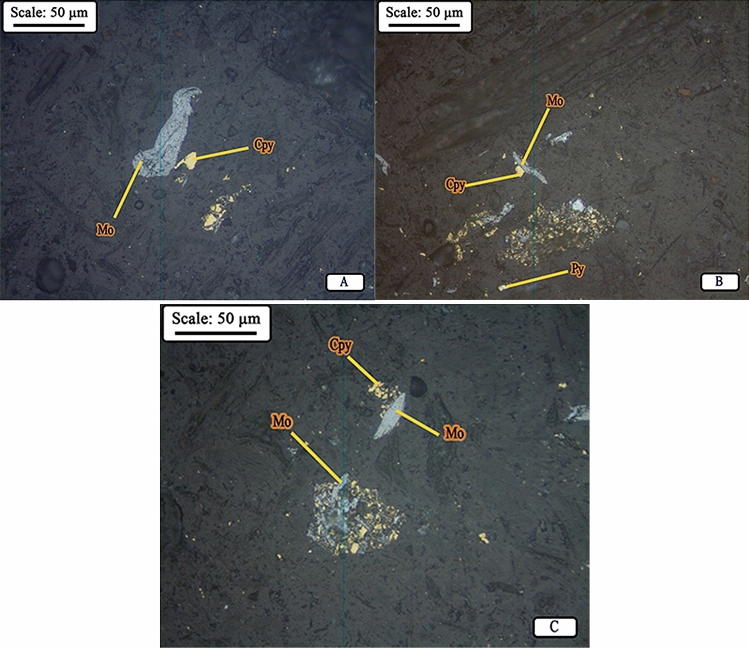
**(A)** Molybdenite liberated particle in feed, **(B)** Distribution of minerals in the product, and **(C) **Molybdenite liberated particles in the product of open-circuit ball mill (*Mo* molybdenite, *Cpy* chalcopyrite, *Py* pyrite).

According to optical reflected light microscopic studies, chalcopyrite, molybdenite, pyrite, and covellite (Table 2) make up the major part of the composition of the middle thickener underflow or open-circuit mill feed. Liberation studies of molybdenite in the feed of open-circuit mill (Fig. 6A) indicate that this mineral has achieved a proper liberation degree (about 98.0%). Therefore, at this stage, grinding leads to more fine production of molybdenite particles and does not cause a significant change in their liberation degree. The study of PSD of the mill product also confirms this; In other words, at this stage, the particles of molybdenite and copper sulfides have become finer and have turned into slimes.

As can be seen in Fig. 6B, the minerals present in the product of open-circuit mill, in the order of abundance, include molybdenite, which is mostly free and has become fine after grinding in mill, liberated chalcopyrite particles (about 32.0%) and interlocked with pyrite and other associated gangue minerals, and liberated and interlocked particles of pyrite. Liberation studies for chalcopyrite and pyrite in the product of mill shows 85.0% and 85.40% values of liberation degree for these minerals, respectively. It can be stated that these two minerals have the same liberation degree. On the other hand, there was no significant interlocking between copper sulfide minerals and molybdenite (Fig. 6C). Coarse particles of molybdenite are observable in some cases, but in general the molybdenite particles are ground to very fine size ranges (slime range).

**Figure 7:**
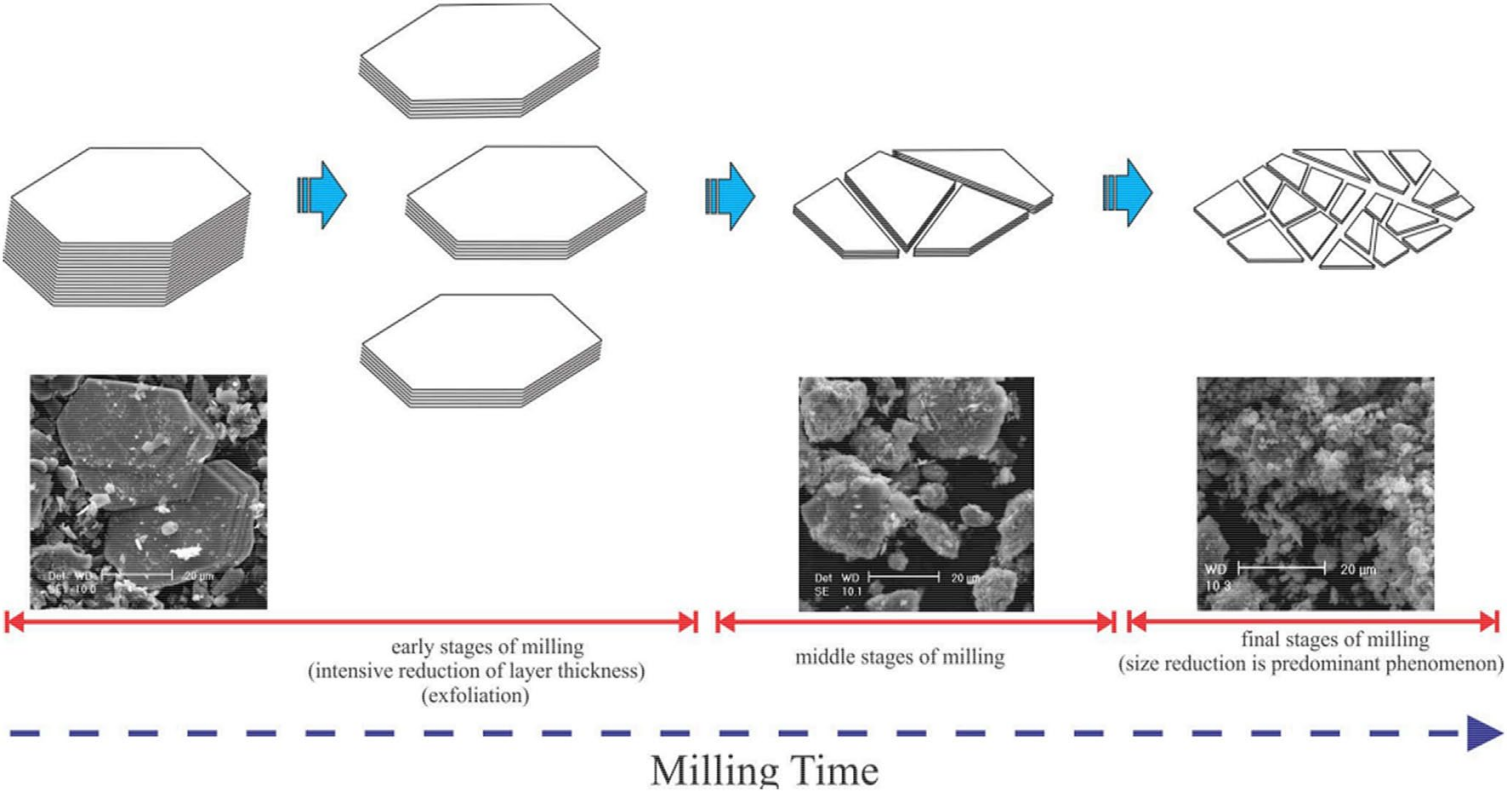
Microscopic image and schematic of molybdenite ore grinding during grinding process^13^.

Due to its anisotropic properties, molybdenite behaves differently from other sulfide minerals. SEM–TEM images (Fig. 7) show how MoS_2_ breaks (cleaves) in layers. Because in molybdenite with hexagonal structure, the S–Mo–S layers are connected with the covalent bond by the Van der Waals forces. Once molybdenite is ground, its cleavage occurs more easily within the weak Van der Waals forces. Hence, the outer layers of the mineral surface are removed under the influence of shear forces; While the compressive forces in the grinding environment affect the mineral edges and cause breakage in the direction of the edges. The above-mentioned grinding processes, which reduce the thickness of the mineral, occur in the early stages of grinding. As the grinding time increases and in the final stages of grinding, the particle size of the produced layers decreases at a slower rate when compared to the early stages of grinding^13^. As the size of the molybdenite particles decreases, the surface-to-edge ratio decreases, resulting in an increase in its hydrophilic properties, which reduces its floatability.

### Investigating the efficiency of closed-circuit ball mill from process mineralogy perspective

#### (A) Investigating the variations in size distribution of the closed-circuit mill feed and product

**Figure 8:**
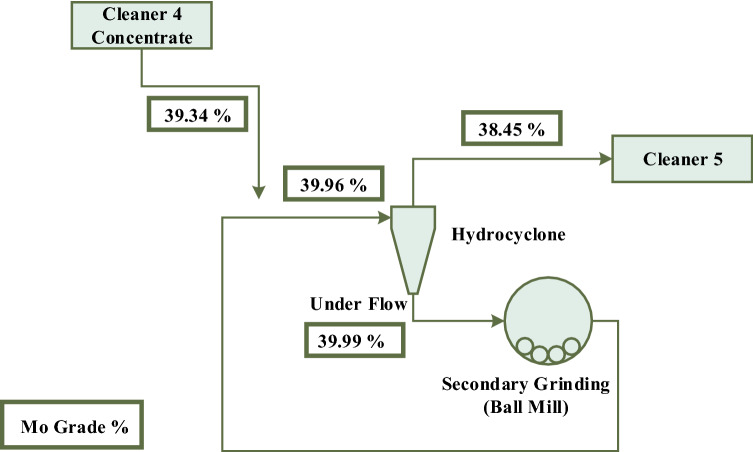
Molybdenite grinding circuit—closed-circuit ball mill.

**Figure 9:**
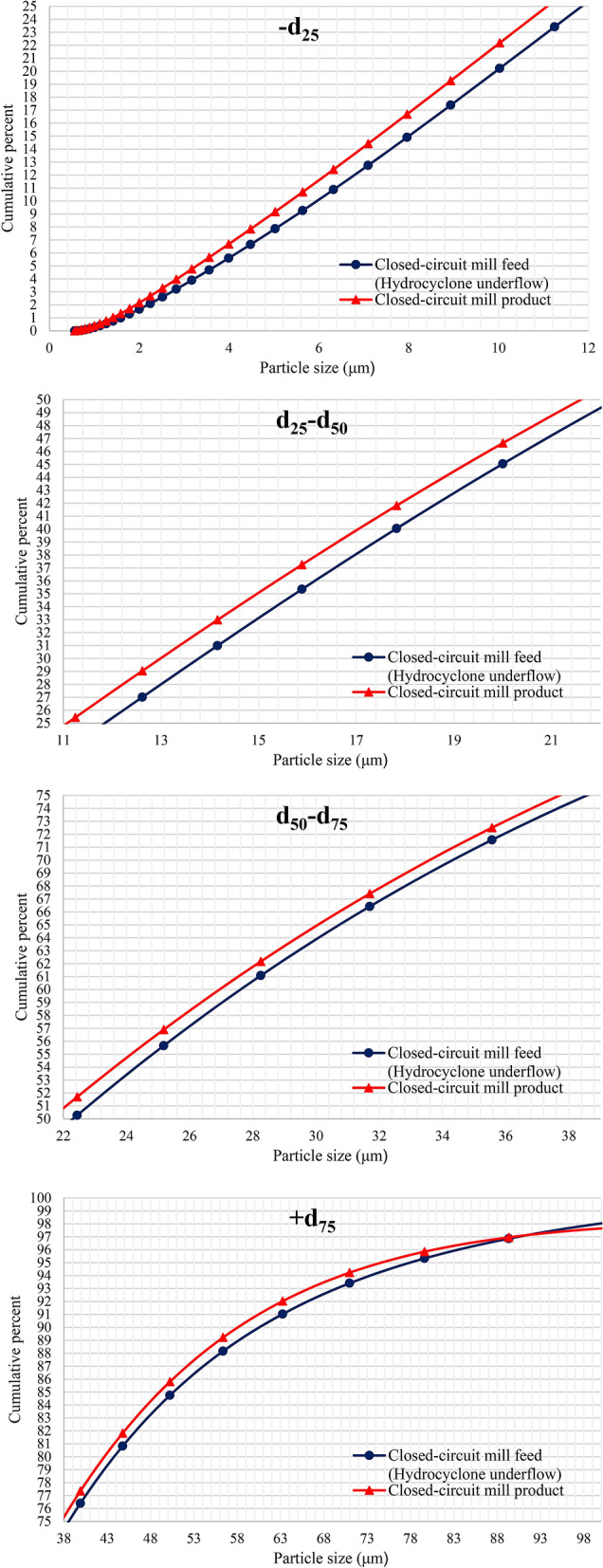
Size distribution diagrams of closed-circuit ball mill feed and product.

The cleaner 4 concentrate with a molybdenum grade of 39.34% and a copper and iron grade of 4.94% and 10.34%, respectively, must be re-floated in order to achieve a higher grade (Fig. 2). In this regard, this concentrate is introduced into the hydrocyclone with a separation limit of 38.0 microns, and the underflow is rejected to the closed-circuit ball mill for regrinding (Fig. 8). The goal of the secondary grinding stage is to achieve the maximum liberation degree of molybdenite and copper minerals from each other. Examination of the hydrocyclone underflow size distribution (ball mill feed) and mill product (Fig. 9 and Table 1) shows that grinding did not have much effect on reducing particle size. In general, the highest grinding occurred in the size range of feed d_25_–d_50_ values.

#### (B) Mineralogy of closed-circuit ball mill feed and product

**Figure 10:**
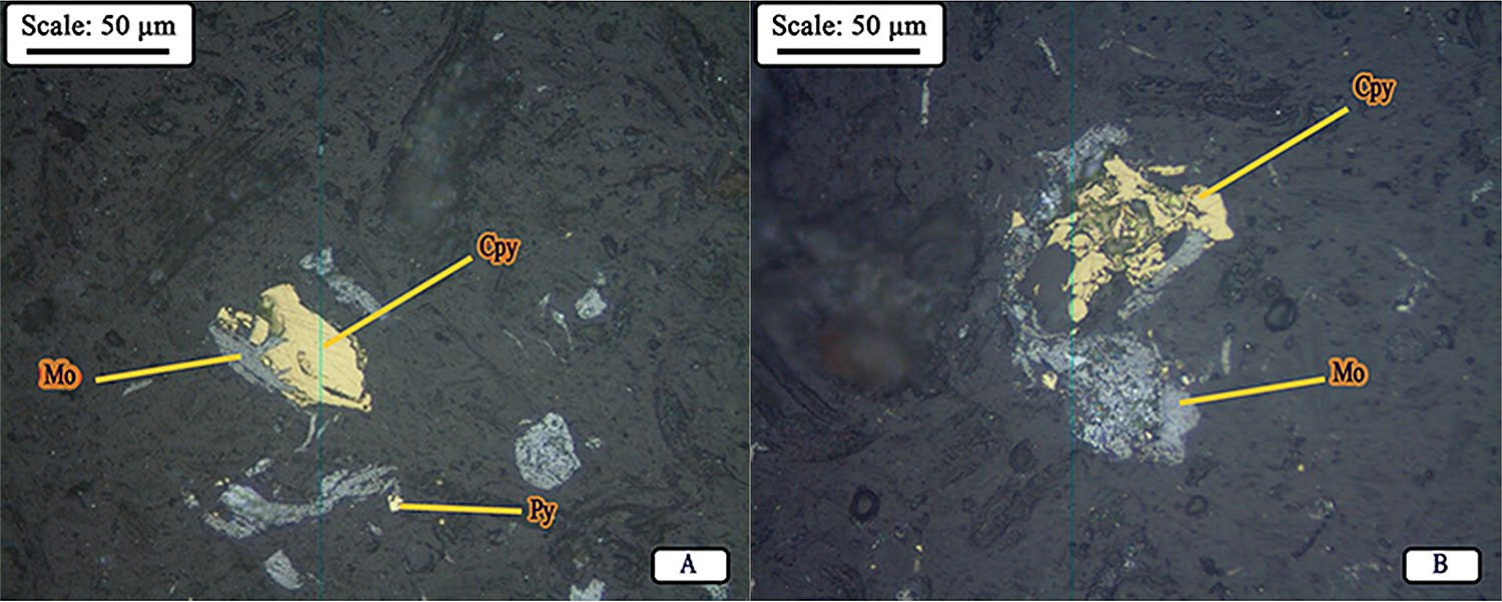
Distribution of minerals in **(A)** feed (hydrocyclone underflow) and **(B)** product of closed-circuit ball mill (*Mo* molybdenite, *Cpy* chalcopyrite, *Py* pyrite).

**Figure 11:**
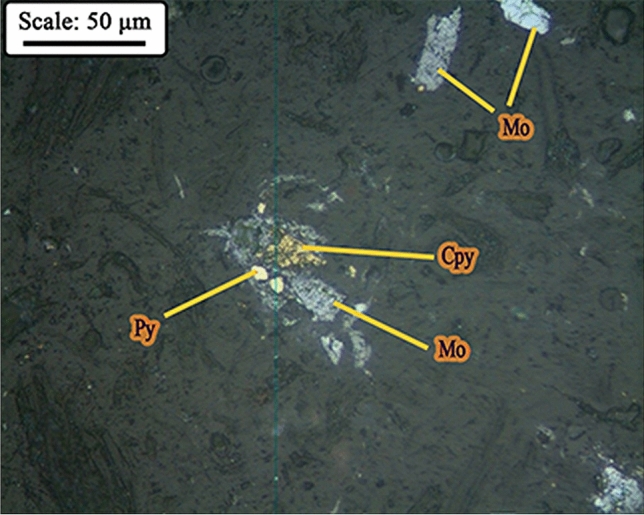
Image of liberated particles of molybdenite in the product of closed-circuit ball mill (*Mo* molybdenite, *Cpy* chalcopyrite, *Py* pyrite).

Microscopic studies have been performed on mill feed (hydrocyclone underflow) and product. Figure 10A,B shows a picture of the minerals distribution in the studied samples. According to studies, the composition of hydrocyclone underflow (mill feed), in order of abundance, includes liberated molybdenite in the form of blades and polygonal fragments (Fig. 11), liberated and interlocked chalcopyrite with gangue minerals and liberated and interlocked pyrite particles. The liberation degree of chalcopyrite and pyrite in mill product are 84.40% and 91.40%, respectively. It is worth mentioning that despite the performed grinding, interlocking between molybdenite and chalcopyrite have rarely been observed. In general, according to mineralogical studies and PSD of feed and product of closed-circuit ball mill, the minerals in feed and product are almost similar in terms of particle liberation degree and only particle size has become finer.

**Figure 12:**
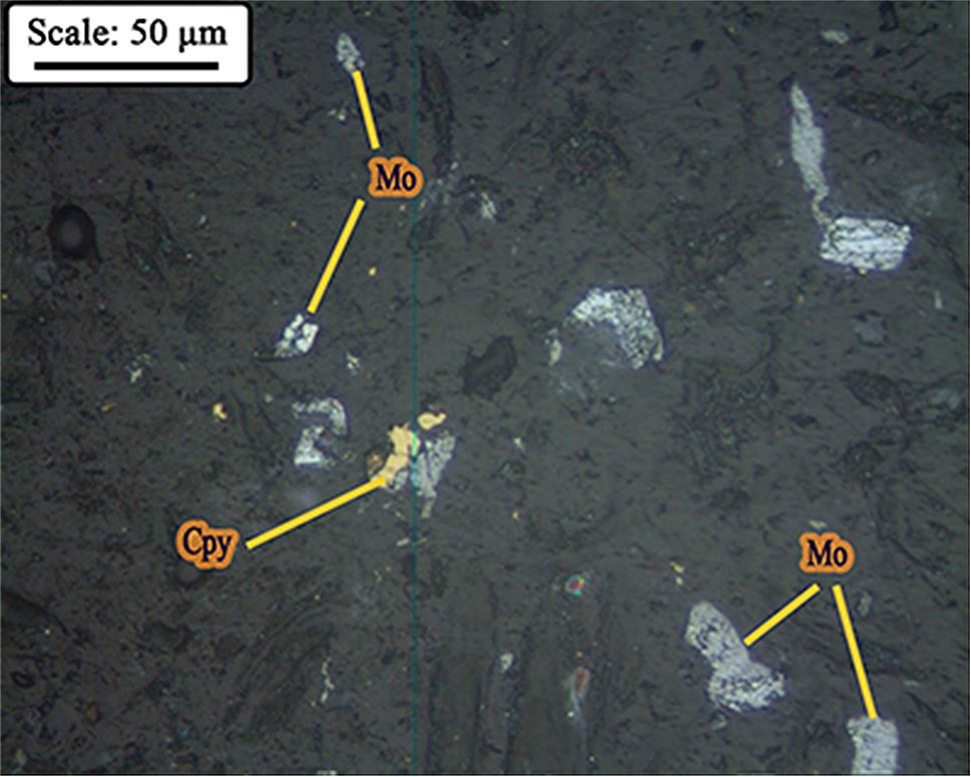
Presence of fine particles in underflow of the hydrocyclone (closed-circuit mill feed)—Sungun copper–molybdenum complex (*Mo* molybdenite, *Cpy* chalcopyrite, *Py* pyrite).

**Figure 13:**
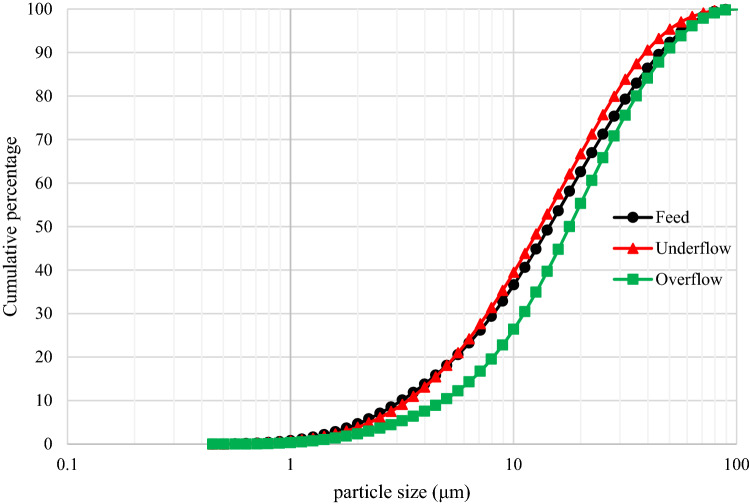
PSD of feed, overflow and underflow of hydrocyclone of molybdenum plant, Sungun copper–molybdenum complex.

As mentioned, the cut size of hydrocyclone in closed circuit with the ball mill is 38.0 microns, however, according to the microscopic images of the hydrocyclone underflow sample (Fig. 12), small particles are also observed in this sample, which is due to the inefficiency of the hydrocyclone. Figure 13 shows the particle size distribution diagram for hydrocyclone feed, overflow, and underflow, which indicates poor classification performance of the hydrocyclone in the circuit, in the separation of fine particles. According to PSD diagram, d_90_ value of feed, overflow and underflow of hydrocyclone are 62.0, 55.0 and 68.0 microns, respectively. On the other hand, by measuring the solid percentage of feed (12.0%) and underflow (15.0%) of hydrocyclone, used with the aim of dewatering and particle size control, it can be concluded that this device did not have a good dewatering performance ^18^. Given the above, the low solid percentage of feed and the abundance of fine particles in the feed can be considered factors responsible for the poor performance of the mill.

### Investigating the effect of presence and absence of closed-circuit ball mill on the grade distribution of molybdenum processing plant concentrate

**Figure 14:**
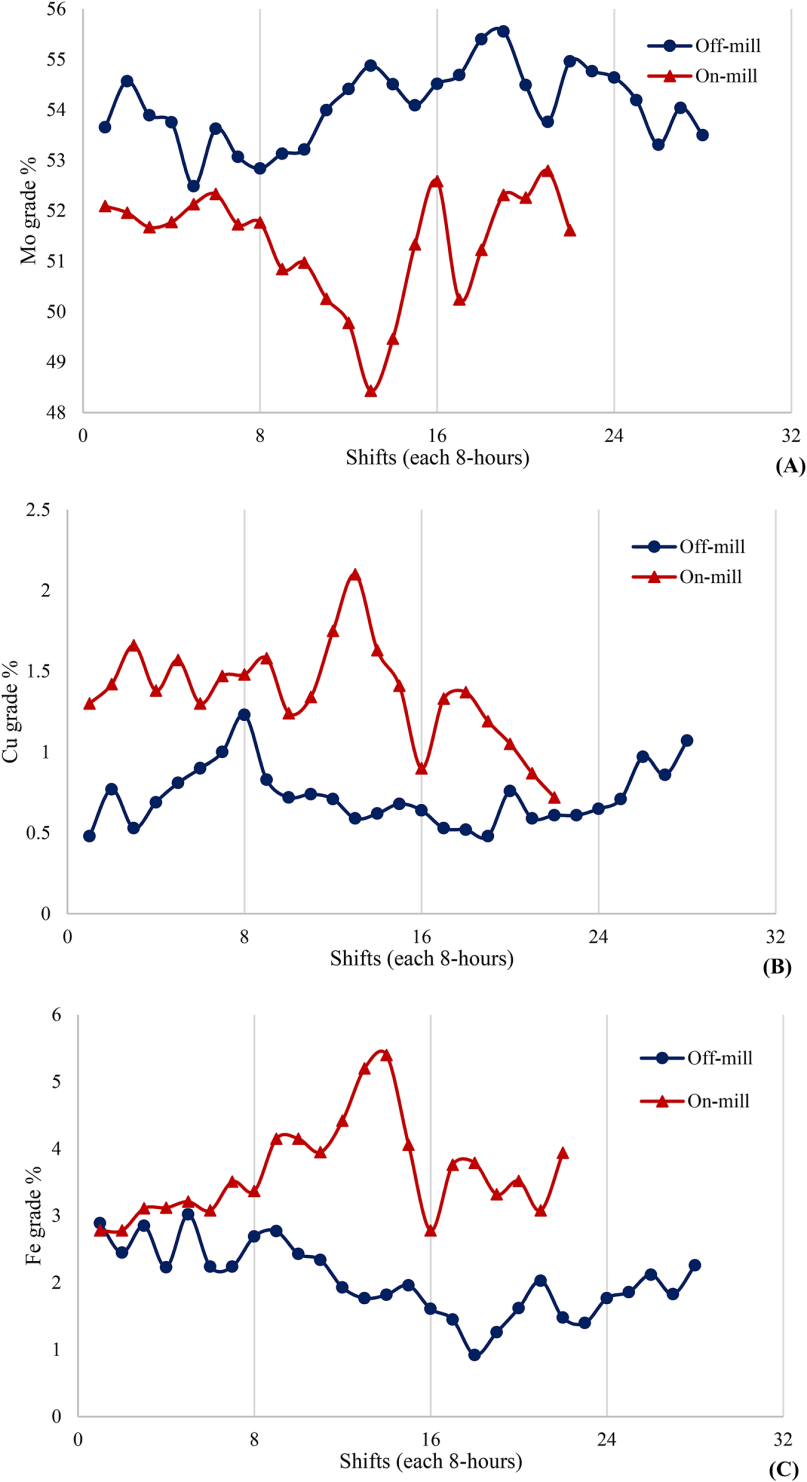
Grade analysis of the final molybdenum concentrate for elements **(A)** molybdenum, **(B)** copper and **(C)** iron in the off and on mode of closed-circuit ball mill in a period of 10 days (every day includes 3, 8-h shifts).

In order to more accurately examine the performance of closed-circuit mill, the results of the final molybdenum concentrate analysis were studied in two modes of mill-on and mill-off in a 10-day period (each day including three 8-h shifts). Figure 14 shows diagrams for grade analysis of molybdenum, copper, and iron in two modes of mill-on and mill-off for closed-circuit ball mill. According to the figure, the plant circuit is operating in optimal conditions when the mill is switched off. As the circuit became out of optimal conditions, the closed-circuit mill switched on, but the start-up of the closed-circuit mill did not improve the conditions of the processing circuit. It is worth mentioning that the optimal condition means the grade of molybdenum, copper and iron in molybdenum concentrate is more than 50.0% and less than 1.0% and 3.0%, respectively (according to the standards of commercial markets). According to the results, the average grade of molybdenum, copper and iron in molybdenum concentrate in mill-on (non-optimal) mode is 51.21%, 1.32% and 3.95%, respectively. While the average values for Mo, Cu and Fe grade in mill-off mode are 53.83%, 0.71% and 2.04%, respectively. As can be seen, in mill-off mode and optimal conditions, grade standards for molybdenum, copper and iron are available in molybdenum concentrate. However, in non-optimal conditions, starting of the mill has no effect on the optimization of these values, and this indicates the inefficiency of the mill.

## Conclusion

It is important to know the type and properties of the minerals in the ore being processed in order to design and optimize the circuit of a processing plant. In this study the efficiency of grinding was investigated by studying the mineralogical properties of feed and product streams to the grinding circuits in the molybdenum processing plant. Analysis of particle size distribution for open and closed-circuit ball mills feed and product showed that d_90_ value of feed and product of open-circuit mill is 43.83 and 42.33 microns, respectively, and d_90_ value of feed and product of closed-circuit mill were 67.95 and 65.13 microns. The performed liberation study also shows that in the feed and product of the open-circuit mill, the liberation degree of molybdenite is almost the same and about 98.0%. Therefore, in the milling stage, the molybdenite particles are only ground and there is no significant change in their liberation degree. On the other hand, because there is no controlling equipment for particle size in open-circuit mill, fine materials turn into slimes and due to the slime coating, entrainment and less efficient collision of the particles with the air bubble, the flotation rate and the grade is reduced. With excessive grinding of materials in the mill, the surface-to-edge ratio, especially in the case of molybdenite, is reduced, and due to reduced hydrophobicity and floatability, fine molybdenite particles are introduced to tailing product or copper concentrate. In the case of closed-circuit mill, the minerals present in the feed and product of the mill are almost identical in terms of particle liberation degree, and only the particle size gets finer. An examination of the molybdenum, copper and iron grade changes over a 10-day period for both mill on and off modes of closed-circuit mill showed that in the mill-off mode, the plant circuit is in optimal conditions (molybdenum, copper and iron grade in the molybdenum concentrate were more than 50.0% and less than 1.0% and 3.0%, respectively), but as the circuit gets out of optimal condition, the start-up of closed-circuit mill has not had an effect on improving the circuit and creating optimal conditions.
